# Regulation of the Cortisol Axis, Glucagon, and Growth Hormone by Glucose Is Altered in Prediabetes and Type 2 Diabetes

**DOI:** 10.1210/clinem/dgad549

**Published:** 2023-09-14

**Authors:** Martin H Lundqvist, Maria J Pereira, Kristina Almby, Susanne Hetty, Jan W Eriksson

**Affiliations:** Clinical Diabetology and Metabolism, Department of Medical Sciences, Uppsala University, 751 85 Uppsala, Sweden; Clinical Diabetology and Metabolism, Department of Medical Sciences, Uppsala University, 751 85 Uppsala, Sweden; Clinical Diabetology and Metabolism, Department of Medical Sciences, Uppsala University, 751 85 Uppsala, Sweden; Clinical Diabetology and Metabolism, Department of Medical Sciences, Uppsala University, 751 85 Uppsala, Sweden; Clinical Diabetology and Metabolism, Department of Medical Sciences, Uppsala University, 751 85 Uppsala, Sweden

**Keywords:** type 2 diabetes, counter-regulatory hormones, glucagon, cortisol, ACTH, insulin resistance

## Abstract

**Context:**

Insulin-antagonistic, counter-regulatory hormones have been implicated in the development of type 2 diabetes (T2D).

**Objective:**

In this cross-sectional study, we investigated whether glucose-dependent regulation of such hormones differ in individuals with T2D, prediabetes (PD), and normoglycemia (NG).

**Methods:**

Fifty-four individuals with or without T2D underwent one hyperinsulinemic-normoglycemic-hypoglycemic and one hyperglycemic clamp with repeated hormonal measurements. Participants with T2D (n = 19) were compared with a group-matched (age, sex, BMI) subset of participants without diabetes (ND, n = 17), and also with participants with PD (n = 18) and NG (n = 17).

**Results:**

In T2D vs ND, glucagon levels were higher and less suppressed during the hyperglycemic clamp whereas growth hormone (GH) levels were lower during hypoglycemia (*P* < .05). Augmented ACTH response to hypoglycemia was present in PD vs NG (*P* < .05), with no further elevation in T2D. In contrast, glucagon and GH alterations were more marked in T2D vs PD (*P <* .05).

In the full cohort (n = 54), augmented responses of glucagon, cortisol, and ACTH and attenuated responses of GH correlated with adiposity, dysglycemia, and insulin resistance. In multilinear regressions, insulin resistance was the strongest predictor of elevated hypoglycemic responses of glucagon, cortisol, and ACTH. Conversely, fasting glucose and HbA1c were the strongest predictors of low GH levels during hypoglycemia and elevated, i.e. less suppressed glucagon levels during hyperglycemia, respectively. Notably, adiposity measures were also strongly associated with the responses above.

**Conclusions:**

Altered counter-regulatory hormonal responses to glucose variations are observed at different stages of T2D development and may contribute to its progression by promoting insulin resistance and dysglycemia.

There is a strong link between obesity and type 2 diabetes (T2D); obesity increases the risk of developing T2D 7-fold compared with normal weight (1) and the vast majority of patients with T2D are overweight or obese (2, 3). The link between obesity and T2D is considered to be primarily mediated via insulin resistance. Several causal mechanisms between obesity and insulin resistance have been suggested, including accumulation of ectopic fat in key metabolic organs (4), hypersecretion of cytokines from inflamed excess adipose tissue (5), and alterations in gut microbiota (6). However, there are still features in the development of T2D that are incompletely understood, which has encouraged the formulation of theories that reach beyond the realm of insulin secretion by the pancreas and insulin action in target tissues.

The bihormonal hypothesis, proposed by R. H. Unger in 1975, highlights the importance of hyperglucagonemia in the development of T2D (7) and has been supported by subsequent translational research in humans and animals (8). Disturbances in the hypothalamic-pituitary-adrenal (HPA) axis, wherein pituitary release of adrenocorticotropic hormone (ACTH) stimulates adrenal secretion of cortisol, have also been implicated in the development of T2D, and this has also been proposed to play a role in the association between psychiatric conditions and T2D (9). Glucagon and cortisol, along with catecholamines and growth hormone (GH), are counter-regulatory hormones that are secreted in response to hypoglycemia, in a process that is coordinated from the brain and brainstem. The combined metabolic effect of these counter-regulatory hormones is to elevate glucose levels, essentially by effects opposite to those of insulin. There is much evidence from animal and human studies that the brain plays a key role in systemic glucose homeostasis and the development of T2D, largely mediated via the regulation of counter-regulatory hormones and the autonomic nervous system (10, 11). Thus, the effects of components of neuroendocrine pathways are well known, but their potential role among individuals developing T2D are not well characterized (10, 11).

Using clamp techniques, our research group has demonstrated that individuals with overweight and insulin resistance have an accentuated HPA axis response to hypoglycemia and an impaired reduction of glucagon during hyperglycemia (12). Going further, we have recently applied population analysis of pooled data from different clamp studies to demonstrate that individuals with insulin resistance have an upward shifted glycemic setpoint for activation of the HPA axis response, situated within the normal glycemic range. These individuals also have generally elevated glucagon levels over the entire glycemic range (13). Conversely, bariatric surgery leads to subacute and persistent attenuation of several counter-regulatory responses to hypoglycemia (14, 15). Taken together, altered glucose-dependent responses of both glucagon and the HPA axis seem to be features of overweight and insulin resistance that potentially may contribute to the development of T2D.

The aim of this study was to investigate if these properties of hormonal counter-regulation, observed in overweight and insulin resistant individuals without T2D, are even more evident in individuals with established T2D. We have therefore examined individuals with T2D and compared the hormonal responses to glucose variations with those of matched individuals without T2D. In addition, groups with normoglycemia or prediabetes were included to elucidate stages of diabetes development. To our knowledge, this is the first study to address contribution of neuroendocrine counter-regulation in T2D development by using normo-, hypo-, as well as hyperglycemic clamps.

## Materials and Methods

### Participants

This cross-sectional study took place at the Uppsala University Hospital and the Department of Medical Sciences at Uppsala University between March 2018 and August 2022. The previously reported cohort (12) was expanded by adding a group of patients with T2D (n = 19) and also by including additional individuals without diabetes (n = 35 in total). Eligibility criteria, in summary, were age 18 to 70 years, body mass index (BMI) 18.5 to 50 kg/m^2^. For participants with T2D, eligibility included diagnosis according to World Health Organization (WHO) criteria with a duration of less than 8 years with glycated hemoglobin (HbA1c) < 70 mmol/mol, no medication or only metformin, and fasting glucose 5.0 to 10.0 mmol/L on the day of the experiments. Detailed inclusion and exclusion criteria and recruitment methods were previously reported (12).

In the main comparison, the T2D group was compared to a subset of participants without diabetes (n = 17, group ND) that were matched on group basis (age, sex, BMI). For auxiliary analyses, the full cohort of participants without diabetes (n = 35) were stratified by glycemic status into prediabetes (PD, n = 18) and normoglycemia (NG, n = 17). PD was defined as a fasting glucose ≥ 5.6 mmol/L (mean of 2 measurements) or HbA1c ≥ 39 mmol/mol (first visit), using American Diabetes Association criteria. Clinical characteristics in all groups are provided in Table 1. Further, the full cohort was also stratified by M-value tertiles.

**Table 1.** Clinical characteristics

Variable	T2D, n = 19	Matched ND, n = 17 (13 PD, 4 NG)	PD, n = 18	NG, n = 17
Age, years	**55 (46; 60)** ^‡^	52 (39; 57)	51 (36; 55)	37 (28; 52)
Sex, m:f	7:12	6:11	8:10	3:14
Weight, kg	**97.5** (**87.9; 115.6)**^‡^	101.9 (85.2; 125.1)	**92.8** (**82.9; 116.7)**^‡^	78.4 (62.5; 88.0)
BMI, kg/m^2^	**36.7** (**29.0; 40.2)**^‡^	32.3 (28.6; 42.0)	**31.1** (**27.5; 39.1)**^‡^	24.4 (22.6; 29.5)
Waist-hip ratio	**0.99 (0.92; 1.05)** ^‡^	0.96 (0.90; 1.00)	**0.97** (**0.90; 1.01)**^‡^	0.85 (0.80; 0.90)
Body fat, %	**41.0** (**34.7; 48.2)**^‡^	42.0 (27.8; 48.6)	37.0 (25.1; 46.0)	28.7 (21.6; 40.7)
Resting heart rate, bpm	**72** (**65; 80)**^‡^	68 (60; 72)	67 (58; 73)	62 (58; 70)
Systolic BP, mmHg	**142** (138; 153)^*†‡^	128 (121; 136)	**128** (**120; 136)**^‡^	115 (111; 127)
Diastolic BP, mmHg	**90** (**85; 95)**^‡^	84 (79; 90)	86 (78; 90)	78 (75; 83)
Fasting plasma glucose, mmol/L	**7.4** (6.9; 8.3)^*†‡^	5.9 (5.6; 6.2)	**6.0** (**5.8; 6.3)**^‡^	5.3 (5.2; 5.5)
IFCC HbA1c, mmol/mol	**49** (45; 55)^*†‡^	36 (33; 37)	36 (32; 37)	33 (32; 35)
NGSP HbA1c, %	**6.6** (6.3; 7.2)^*†‡^	5.4 (5.2; 5.5)	5.4 (5.1; 5.5)	5.2 (5.1; 5.4)
Serum insulin, pmol/L	**119.8** (**87.2; 173.6)**^‡^	84.0 (64.4; 137.2)	**80.4** (**56.8; 121.6)**^‡^	39.2 (28.3; 64.9)
Serum C-peptide, nmol/L	**1.4** (1.2; 1.9)^*†‡^	1.0 (0.9; 1.4)	**1.0** (**0.8; 1.2)**^‡^	0.6 (0.5; 0.8)
HOMA-IR	**6.0** (3.9; 8.4)^*†‡^	3.2 (2.2; 4.8)	**3.2** (**2.2; 4.8)**^‡^	1.3 (1.0; 2.2)
M-value*^a^*, mg kgFFM^−1^ min^−1^	**3.9** (3.0; 6.4)^*†‡^	6.8 (4.0; 10.6)	**7.6** (**4.9; 10.2)**^‡^	11.6 (9.3; 14.4)
Plasma cholesterol*^b^*, mmol/L	4.0 (3.8; 4.3)	4.5 (3.6; 5.8)	4.5 (3.6; 5.3)	4.4 (3.8; 5.3)
Plasma LDL cholesterol, mmol/L	2.4 (2.0; 2.8)	3.0 (2.3; 4.0)	3.0 (2.3; 3.7)	2.4 (2.0; 2.7)
Plasma HDL cholesterol, mmol/L	1.1 (0.9; 1.4)	1.1 (0.9; 1.4)	1.1 (0.9; 1.4)	1.3 (1.1; 1.8)
Plasma triglycerides, mmol/L	**1.29** (**1.01; 1.83)**^‡^	1.02 (0.72; 1.89)	**1.01** (**0.76; 1.59)**^‡^	0.62 (0.50; 0.75)
Plasma glucagon, ng/L	39.4 (24.5; 50.4)	33.0 (22.3; 41.2)	30.3 (19.8; 40.1)	36.8 (30.7; 44.5)
Serum cortisol, nmol/L	253 (201; 306)	196 (163; 275)	211 (166; 318)	236 (210; 296)
Plasma ACTH, pmol/L	4.1 (2.0; 5.5)	2.9 (2.2; 4.5)	2.8 (2.1; 3.8)	2.5 (2.1; 3.0)
Plasma/serum GH, µg/L	**0.12** (**0.05; 0.33)**^‡^	0.23 (0.04; 0.97)	0.28 (0.06; 1.49)	1.20 (0.09; 4.88)
Metformin, n (%)	**14** (73.7)^*†‡^	0 (0)	0 (0)	0 (0)
Antihypertensive treatment, n (%)	**9** (47.4)^*†‡^	1 (5.9)	1 (5.6)	0 (0)
Lipid-lowering treatment, n (%)	**10 (52.6)** ^* † ‡^	1 (5.9)	1 (5.6)	0 (0)

Data shown for participants with type 2 diabetes (T2D), matched participants without T2D (ND), prediabetes (PD), and normoglycemia (NG), presented as median (IQR) or n (%). First measurement or mean from both visits (glucose, hormones, HOMA-IR). Blood samples were taken at approximately 8:30 Am after an overnight fast. Significant results are **bold**.

Abbreviations: ACTH, adrenocorticotropic hormone; bpm, beats per minute; FFM, free fat mass; GH, growth hormone; HDL, high-density lipoprotein; LDL, low-density lipoprotein;.

^*a*^Data missing for 1 participant in T2D.

^*b*^Data missing for 3 participants in ND, 3 participants in PD, and 4 participants in NG.

^*^ *P* < 0.05, vs ND.

^†^ *P <* 0.05, vs PD.

^‡^ *P* < .05, vs NG.

### Normo-, Hypo-, and Hyperglycemic Clamp Protocols

The study procedures, including clamp designs have been described previously (12). A brief description will follow below, but for details we refer to the previous article.

Each subject underwent one hyperinsulinemic-normoglycemic-hypoglycemic clamp (henceforth denoted *hypoglycemic clamp*) and one hyperglycemic clamp experiment in a randomized order on 2 separate visits with 1 to 5 weeks between. Participants arrived to the clinic in the morning after a fast of at least 10 hours and had not taken any medication on the same morning. Following baseline fasting blood samples and anthropometric assessments, clamps were initiated at approximately 9:00 Am. The hypoglycemic clamp consisted of 3 phases: the normoglycemic phase (0-80 minutes, target glucose 5.0 mmol/L), the hypoglycemic phase (80-185 minutes, sequential target glucose plateaus of 3.8, 3.2, and 2.7 mmol/L) and the recovery phase (185-215 minutes), obtained by simultaneous infusions of glucose 200 mg/mL and insulin 56 mU m^−2^ BSA min^−1^ after priming. If fasting levels were above 7.0 mmol/L, the normoglycemic phase was not initiated until glucose levels were below this limit. M-value, an inverse measure of insulin resistance, was defined as the mean glucose infusion rate in mg × kg fat-free mass (FFM)^−1^× min^−1^ from 40 to 80 minutes of the normoglycemic phase. At 185 minutes, the insulin infusion was stopped, and glucose was raised by a constant infusion rate of 300 mg × kgFFM^−1^ × h^−1^.

In the hypergycemic clamp, glucose levels were first kept at fasting baseline levels for 30 minutes, then a flexible glucose infusion (200 mg/mL) was administered to raise glucose levels stepwise to targets of +3 mmol/L, + 6 mmol/L and +9 mmol/L above baseline levels for 45 minutes each. The glucose infusion was discontinued at 165 minutes and glucose levels were allowed to return to the normal range.

### Clinical Biochemistry

**Figure 1.**
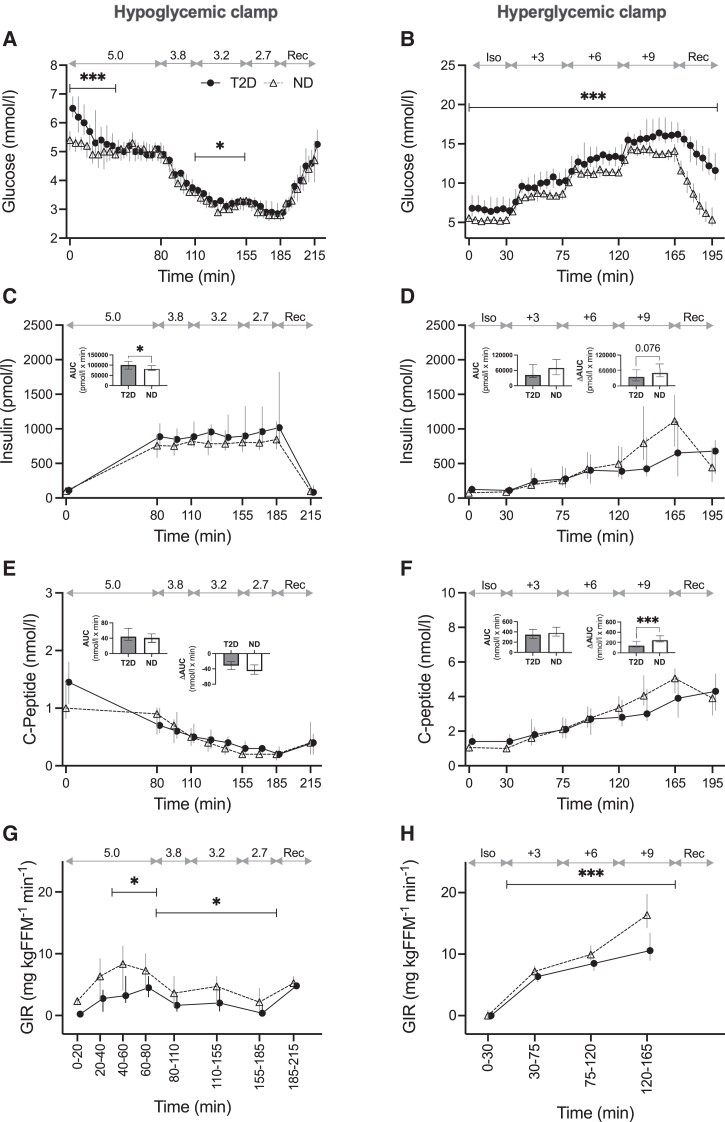
Levels of glucose (bedside glucometer; A, B), insulin (C, D), C-peptide (E, F), and glucose infusion rate (G, H) in the hypoglycemic (left panel) and hyperglycemic (right panel) clamps in participants with type 2 diabetes (T2D, filled circles and solid lines, n = 18 in left panel, n = 19 in right) and without type 2 diabetes (ND, open triangles and dashed lines, n = 17 in left panel, n = 16 in right). Data displayed as median and IQR. Glycemic targets in mmol/L displayed on top of each graph. *P* values refer to groupwise comparisons, by Mann-Whitney U tests, of individual means (A, B, G, H) during indicated intervals, AUC or △AUC during the hypoglycemic (80-185 minutes) or hyperglycemic phase (30-165 minutes). Abbreviations: AUC, area under the curve; FFM, fat free mass; GIR, glucose infusion rate; Iso, isoglycemic phase; Rec, recovery phase. *P < 0.05, ***P < 0.001.

**Figure 2.**
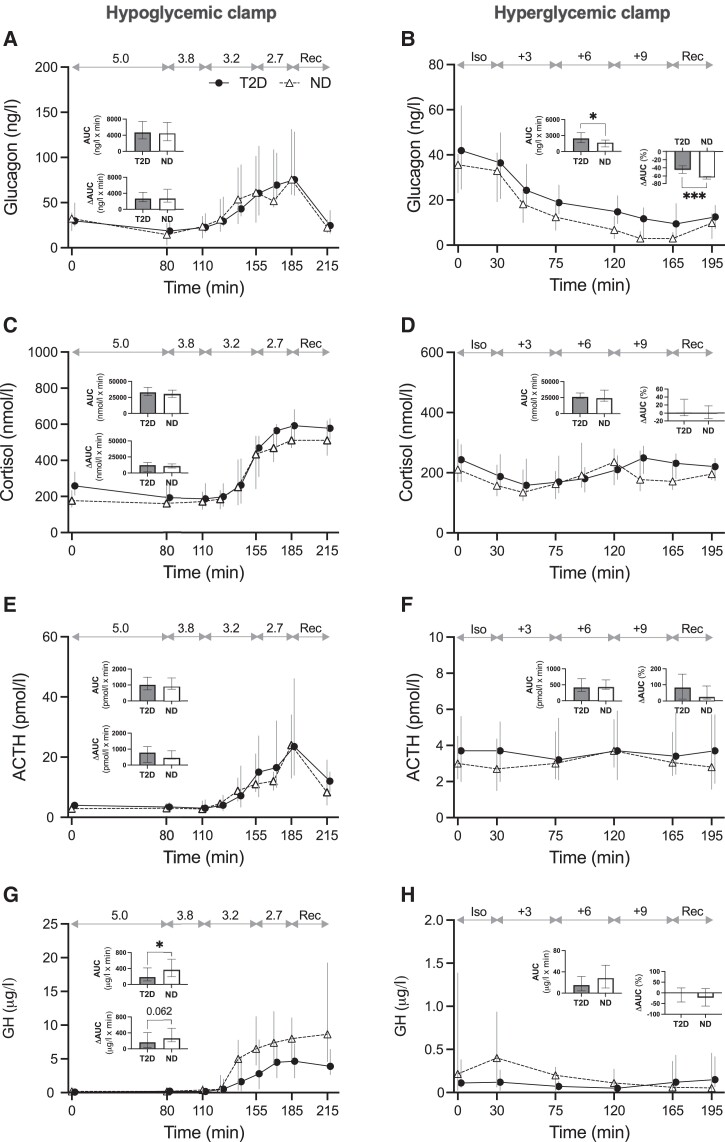
Levels of glucagon (A, B), Cortisol (C, D), ACTH (E, F) and GH (G, H) during hypoglycemic (left panel) and hyperglycemic (right panel) clamps in participants with type 2 diabetes (T2D, filled circles and solid lines, n = 18 in left panel, n = 19 in right) and without type 2 diabetes (ND, open triangles and dashed lines, n = 17 in left panel, n = 16 in right). Data displayed as median and IQR. Glycemic targets in mmol/L displayed on top of each graph. *P* values refer to Mann-Whitney U tests of AUC or △AUC of hormones (in % in right panel) from 80 to 185 minutes (hypoglycemic clamps) or 30 to 165 minutes (hyperglycemic clamps). Abbreviations: ACTH, adrenocorticotropic hormone; AUC, area under the curve; GH, growth hormone; Iso, isoglycemic phase; Rec, recovery phase. *P < 0.05, ***P < 0.001.

Glucose levels were measured bedside every 5 minutes during both clamps with a Contour Glucose Meter (Bayer Healthcare, Leverkusen, Germany). In fasting conditions (approximately 8:30 Am) before the clamps and at regular timepoints during both clamps (displayed in Figs. 1 and 2), blood samples for hormonal analyses were obtained from an arterialized vein and were analyzed within 4 hours or frozen at −80 °C. Serum insulin, cortisol, C-peptide (all CobasE, Roche), plasma ACTH, and plasma/serum GH (both Immulite 2000XPi, Siemens Healthcare Global) were analyzed at the Department of Clinical Chemistry, Uppsala University Hospital. Plasma glucagon was measured with ELISA (#10-1271-01, Mercodia, Uppsala, Sweden, within-assay coefficient of variation (CV) 2.1% to 14%, between-assay CV 7.0% to 16%) at the Clinical Diabetes Research Laboratory. Values below the lower limit of quantification (LLQ) for each analyte were imputed to LLQ/2.

### Statistical Analysis

The sample size was estimated to 15 participants in each group according to power calculations previously undertaken and then confirmed by our previous study (12), assuming a power of 80% to detect a 25% difference in levels of glucagon and cortisol, given an inter-subject CV of 0.20 and a 2-sided α of .05. SPSS for Mac version 28 (IBM corp., Armonk, NY) was used for statistical analysis. Figures were constructed in GraphPad Prism version 9.1.0 (GraphPad Software, San Diego, Ca). Data are presented as median (IQR) unless otherwise stated. For repeated assessments, trapezoidal area under the curve (AUC) and ΔAUC (subtracting the area under the baseline value) during the hypoglycemic and hyperglycemic phases (80-185 and 30-165 minutes, respectively) were used as summary measures. When deemed appropriate, considering group differences at baseline and hormone trajectories during the clamps, ΔAUC was expressed in % relative to the baseline value rather than in absolute terms. Group differences were analyzed with Mann-Whitney U tests, Fisher's exact test (both regarding T2D vs ND), Kruskal-Wallis H test with post hoc pairwise Dunn's test (adjusted by false discovery rate) or Fisher-Freeman-Halton test (both regarding T2D vs PD and NG).

#### Generalized estimating equations

The relationship between hormone levels at the end of each clamp phase (8 samples per individual) and glucose levels in T2D vs ND was analyzed with generalized estimating equations. Subject ID was selected as subject variable and the timepoint of measurement as Within-Subject variable. Type of model was selected as gamma distribution, the robust estimator was selected as covariance structure and working correlation matrix structures as well as link functions were guided by convergence issues and the fit of model predictions. Glucose (mean during the preceding 20 minutes for each timepoint) and group (ND = 0; T2D = 1) as well as the interaction glucose*group were selected as model effects. Glucose and group were centered to the grand mean before analysis.

#### Correlation and multilinear regression analyses in full cohort

Spearman's rank correlation analyses were computed between hormonal responses (AUC and ΔAUC) and metabolic parameters in the full cohort (n = 54) as well as stratified by glycemic status (T2D/PD/NG).

For multilinear regression analyses, metabolic parameters were clustered into markers of adiposity (BMI, waist-hip ratio, body fat %), glycemic control (fasting glucose, HbA1c) and insulin resistance (M-value, homeostatic model assessment for insulin resistance [HOMA-IR]). Within each cluster, only one variable was included in the same model. M-value was used as the primary measure of insulin resistance. Only variables with a Spearman's rank correlation *P* value < .10 were included in the models, and models were only generated for each respective outcome variable if such variables were found in more than one cluster. Additional models were explored in case of *P <* .10 for more than one variable within the same cluster. The best fitting model for each outcome variable, as assessed by the adjusted *R^2^*, was considered the final model and this model was further adjusted for age and sex (female = 0, male = 1). Fulfillment of assumptions was assessed by visual inspection of data, including histograms as well as pp-plots of residuals and homoscedasticity. Outliers and leverage points were generally tolerated if not deemed influential as determined by Cook's distance (<1). In case of violation of assumptions, transformation of the dependent variable by the square root, logarithm base 10, or combinations of these transformations were carried out after appropriate normalization of the data.

### Ethics

All study procedures were performed in accordance with the Declaration of Helsinki. Ethical approval was granted by the local Research Ethics Committee of Uppsala (DNR 2017/550) with amendments approved by the Swedish Ethical Review Authority (DNR 2019-04166, 2021-05714-02). Participants were informed about the study verbally and in writing and signed an Informed Consent Form.

## Results

All included participants completed the study, except one subject in T2D, who did not complete the hypoglycemic clamp for personal reasons and one subject in ND, who did not complete the hyperglycemic clamp due to difficulties in achieving venous access.

### Group Comparison of Clamp Assessments in T2D vs ND

Glucose levels were slightly higher in T2D vs ND during the first 40 minutes of the normoglycemic phase and the intermediate stage of the hypoglycemic phase (target glucose 3.2 mmol/L) and during all stages of the hyperglycemic clamp (Fig. 1A and 1B). Insulin AUC was higher in T2D vs ND during the hypoglycemic phase, suggesting impaired insulin clearance, but it did not differ during the hyperglycemic phase, although levels were clearly lower in T2D at the end of this phase, resulting in a borderline significant difference in ΔAUC (Fig. 1C and 1D). While C-peptide AUC did not differ between the groups in both clamps, C-peptide ΔAUC was lower in T2D vs ND in the hyperglycemic clamp but not in the hypoglycemic clamp (Fig. 1E and 1F), in congruency with the insulin trajectory and indicative of beta-cell dysfunction. Glucose infusion rate was lower in T2D vs ND in both clamps (Fig. 1G and 1H) and, accordingly, so was the M-value.

**Figure 3.**
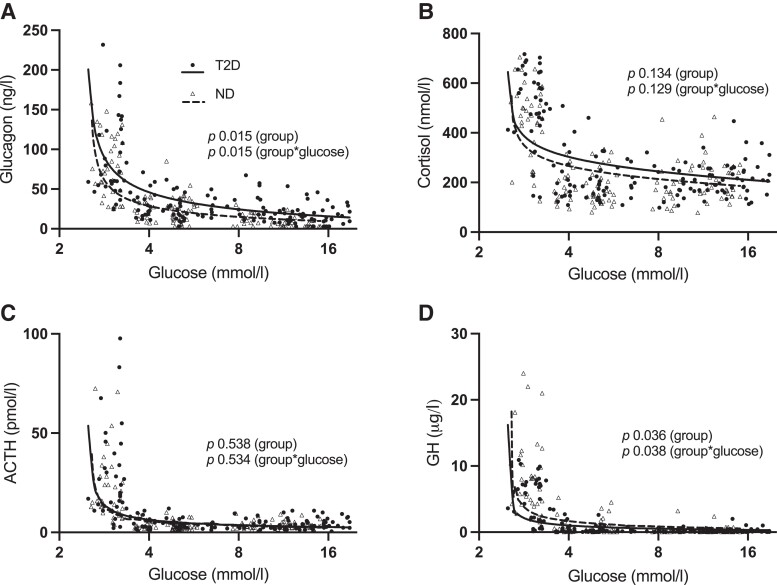
Levels of glucagon (A), cortisol (B), ACTH (C), and GH (D) at the end of each clamp stage (8 samples per individual) compared to individual mean glucose levels during the preceding 20 minutes. Log2 scale on x-axis. Datapoints represent individual values for participants with type 2 diabetes (T2D, n = 19, filled circles) and without type 2 diabetes (ND, n = 17, open triangles). Curves represent predicted group means by generalized estimating equations for T2D (solid) and ND (dashed), interpolated with cubic splines. *P* values refer to results from generalized estimating equations. Matrix structure was AR1 (A) or Independent (B-D). Link function was power of −2 (A, D), −6 (B), and −5 (C). ACTH levels were log-transformed (base 10) prior to analysis. group = estimate of group effect, group*glucose = estimate of interaction between group and glucose. Abbreviations: ACTH, adrenocorticotropic hormone; GH, growth hormone.

Glucagon AUC and ΔAUC did not differ between the groups in the hypoglycemic clamp, but both were higher in T2D vs ND in the hyperglycemic clamp (Fig. 2A and 2B). Cortisol and ACTH did not differ between the groups in both clamps (Fig. 2C-2F). The GH AUC was lower in T2D vs ND in the hypoglycemic clamp, but neither GH AUC nor ΔAUC differed between the groups in the hyperglycemic clamp (Fig. 2G and 2H). Fig. 3 displays levels of counter-regulatory hormones at the end of each clamp stage in relation to individual mean glucose levels during the preceding 20 minutes. Glucagon levels were higher in the normo-hyperglycemic range in T2D vs ND (Fig. 3A). In contrast, there was no clear group difference during hypoglycemia. Cortisol and ACTH did not differ between the groups (Fig. 3B and 3C). GH was generally lower in T2D vs ND (Fig. 3D).

### Subgroup Comparisons of Hormonal Responses by Glycemic Stage and M-Value Tertile

**Figure 4.**
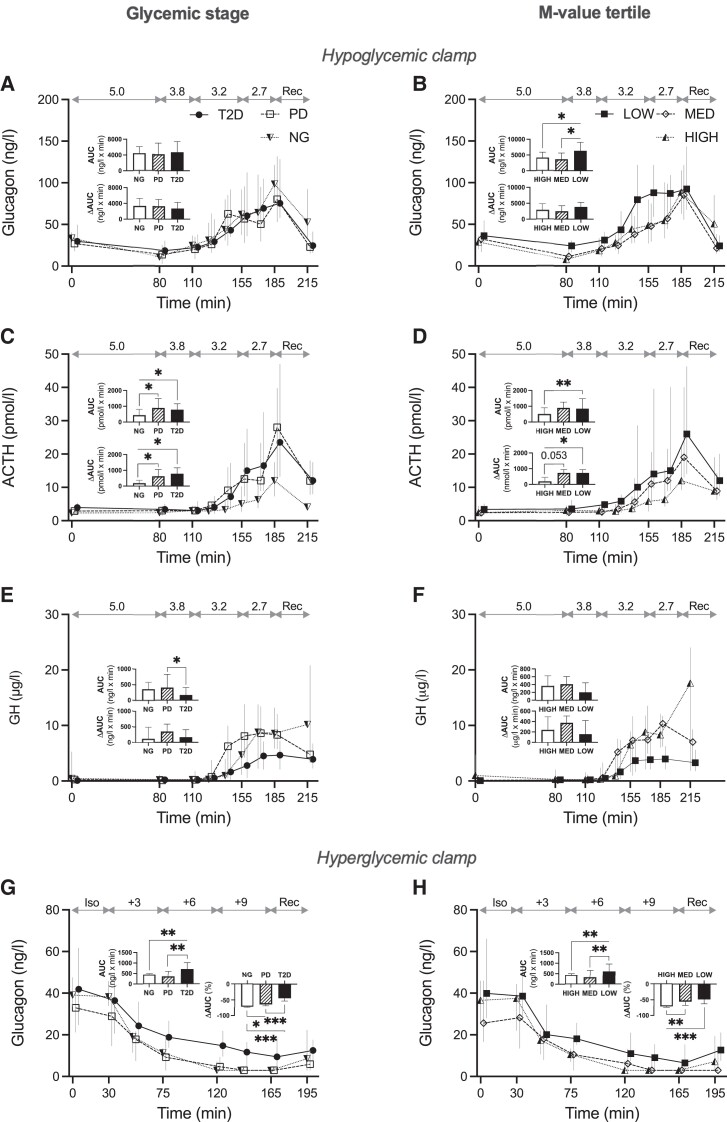
Levels, AUC, and △AUC of glucagon (A, B, G, H), ACTH (C, D), and GH (E, F) during hypoglycemic (A-F) and hyperglycemic clamps (G, H) by glycemic stage according to American Diabetes Association criteria (left panel) and tertiles of M-value (right panel). △AUC in % in hyperglycemic clamp (G, H). Data presented as median and IQR. Glycemic targets in mmol/L displayed on top of each graph. NG = normoglycemia (semi-filled triangles, dashed lines and open bars, n = 17 A, C, E, n = 16 G), PD = prediabetes (open diamonds, dashed lines and diagonally striped bars, n = 18), T2D = type 2 diabetes (filled circles, solid lines and filled bars, n = 18 A, C, E, n = 19 G), High = M-value ≥9.7 mg kgFFM^−1^ min^−1^(open triangles, dashed lines and open bars, n = 18), MED = M-value 5.1-9.7 mg kgFFM^−1^ min^−1^ (filled triangles, dashed lines, diagonally striped bars, n = 17), LOW = M-value ≤5.1 mg kgFFM^−1^ min^−1^ (filled squares, solid lines and filled bars, n = 17 B, D, F, n = 16 H). Abbreviations: ACTH, adrenocorticotropic hormone; AUC, area under the curve; GH, growth hormone; Rec, recovery phase. *P < 0.05, **P < 0.01, ***P < 0.001.

In the hypoglycemic clamp, glucagon responses did not differ depending on glycemic status in the full cohort (Fig. 4A). ACTH responses were elevated in T2D and PD vs NG with no difference between PD and T2D (Fig. 4C, similar but non-significant result for cortisol responses, data not shown). GH AUC was lower in T2D vs PD (Fig. 4E). In the hyperglycemic clamp, glucagon AUC was higher in T2D compared to both PD and NG, while glucagon suppression (ΔAUC) was different between all 3 groups, with gradually more marked attenuation from NG to PD to T2D (Fig. 4G). The GH AUC was lower in T2D vs NG (*P* = .018) but ACTH and cortisol responses did not differ between the groups (data not shown).

The low M-value tertile of the full cohort, i.e., the most insulin resistant, had elevated glucagon AUC compared to the other groups in both clamps (Fig. 4B and 4H). The suppression of glucagon during hyperglycemia was more attenuated in the intermediate compared to the high tertile (Fig. 4H). The ACTH responses to hypoglycemia was higher in the low vs the high tertile (Fig. 4D, similar result for cortisol, data not shown). Cortisol and ACTH hyperglycemic responses did not differ between the M-value tertiles (data not shown). GH AUC was lower in the low vs the high tertile in the hyperglycemic clamp (*P* = .021; data not shown) but GH responses did not differ between the tertiles in the hypoglycemic clamp (Fig. 4F).

### Correlations and Multilinear Regressions

Correlations between hormonal responses and metabolic parameters in the full cohort are displayed in Table 2 and stratified by glycemic status in Tables 3 and 4. The final models, adjusted for age and sex, are displayed in Table 5.

**Table 2.** Spearman's rank correlation coefficients between hormonal responses and metabolic parameters for all participants (n = 53, both clamps)

	Hypoglycemic clamp								Hyperglycemic clamp							
	Glucagon		Cortisol		ACTH		GH		Glucagon		Cortisol		ACTH		GH	
Measure	AUC	ΔAUC	AUC	ΔAUC	AUC	ΔAUC	AUC	ΔAUC	AUC	ΔAUC	AUC	ΔAUC	AUC	ΔAUC	AUC	ΔAUC
BMI	0.03	−0.08	0.20	0.21	**0**.35*	**0**.31*	**−0**.34*	−0.20	**0**.29*	**0**.59*	−0.15	0.16	0.24^†^	**0**.34*	**−0**.33*	0.25^†^
WHR	0.26^†^	0.15	**0**.35*	**0**.41*	**0**.57*	**0**.44*	0.05	**0**.28*	**0**.43*	**0**.39*	−0.15	−0.08	0.23	0.05	**−0**.54*	0.09
Body fat	−0.11	−0.20	0.03	0.02	0.10	0.13	**−0**.44*	**−0**.35*	0.14	**0**.46*	−0.06	0.18	0.08	0.22	−0.15	0.20
Fasting Glucose	−0.03	−0.18	0.23^†^	0.19	**0**.43*	**0**.31*	−0.24^†^	−0.13	**0**.43*	**0**.78*	−0.16	−0.10	0.14	0.15	**−0**.44*	0.23
HbA1c	−0.07	−0.24^†^	0.20	0.03	**0**.30*	0.19	−0.19	−0.18	**0**.48*	**0**.77*	−0.03	−0.03	0.15	**0**.30*	**−0**.31*	0.18
M-value*^a^*	**−0**.35*	−0.14	**−0**.43*	**−0**.31*	**−0**.50*	**−0**.43*	0.22	0.12	**−0**.47*	**−0**.61*	0.04	−0.20	−0.27^†^	−0.09	**0**.43*	−0.14
HOMA-IR	0.27^†^	0.05	**0**.32*	**0**.28*	**0**.47*	**0**.37*	**−0**.35*	−0.18	**0**.56*	**0**.64*	−0.12	0.01	0.31*	0.11	**−0**.52*	0.23^†^

ΔAUC in % in hyperglycemic clamp. Significant coefficients are **bold**.

Abbreviations: ACTH, adrenocorticotropic hormone; AUC, area under the curve; BMI, body mass index; GH, growth hormone; HbA1c, glycated hemoglobin; HOMA-IR, homeostatic model assessment for insulin resistance; WHR, waist-hip ratio.

^*a*^Data missing for 1 subject in the hyperglycemic clamp correlations (total n = 52).

^*^ *P <* .05,

^†^ *P <* .10.

**Table 3.** Spearman's rank correlation coefficients between hypoglycemic hormonal responses and metabolic parameters for all participants (n = 53) stratified by glycemic status

Hypoglycemic clamp												
AUC	Glucagon			Cortisol			ACTH			GH		
Glycemic status	T2D	PD	NG	T2D	PD	NG	T2D	PD	NG	T2D	PD	NG
n	18	18	17	18	18	17	18	18	17	18	18	17
BMI	0.20	−0.05	−0.05	0.02	−0.12	0.05	0.29	−0.10	**0**.51*	**−0**.47*	−0.26	−0.41
WHR	**0**.55*	0.15	0.23	0.34	0.10	0.14	0.14	**0**.53*	**0**.63*	0.30	0.18	−0.18
Body fat	−0.34	−0.05	−0.03	−0.23	−0.14	−0.12	−0.04	−0.30	0.27	**−0**.58*	−0.39	−0.44^†^
Fasting glucose	0.30	**−0**.53*	0.04	0.13	**−0**.55*	0.03	0.19	0.11	0.47^†^	−0.16	−0.26	−0.01
HbA1c	0.41^†^	**−0**.62*	0.37	0.17	−0.14	0.12	0.39	−0.13	0.34	0.11	0.22	−0.03
M-value	−0.41^†^	−0.40	−0.26	−0.29	0.11	**−0**.65*	−0.43^†^	−0.09	**−0**.76*	0.21	**0**.51*	−0.35
HOMA-IR	**0**.60*	0.17	0.29	0.36	−0.33	0.31	**0**.53*	−0.12	**0**.69*	−0.43^†^	**−0**.56*	0.01
**ΔAUC**	Glucagon			Cortisol			ACTH			GH		
BMI	0.38	−0.06	−0.17	0.01	−0.21	0.26	0.26	−0.23	0.44^†^	−0.36	−0.07	−0.31
WHR	**0**.47*	0.25	0.03	**0**.55*	0.20	0.03	0.06	0.41^†^	0.27	0.45^†^	**0**.59*	−0.16
Body fat	−0.17	−0.11	−0.21	−0.39	−0.14	0.00	0.01	−0.25	0.35	**−0**.59*	−0.35	−0.37
Fasting glucose	0.23	**−0**.68*	−0.05	0.05	**−0**.52*	−0.04	−0.04	0.10	−0.01	0.07	−0.29	−0.10
HbA1c	0.24	**−0**.68*	0.10	0.04	−0.30	−0.29	0.21	−0.10	0.06	0.14	−0.07	−0.07
M-value	−0.34	−0.19	−0.13	0.03	0.03	**−0**.50*	−0.32	−0.07	−0.41	0.14	0.30	−0.17
HOMA-IR	**0**.55*	0.04	0.06	0.13	−0.16	**0**.63*	0.27	−0.19	**0**.67*	−0.24	−0.25	−0.01

Significant coefficients are **bold**.

Abbreviations: ACTH, adrenocorticotropic hormone; AUC, area under the curve; BMI, body mass index; GH, growth hormone; HbA1c, glycated hemoglobin; HOMA-IR, homeostatic model assessment for insulin resistance; NG, normoglycemia; PD, prediabetes; T2D, type 2 diabetes; WHR, waist-hip ratio.

^*^ *P <* .05.

^†^ *P <* .10

**Table 4.** Spearman's rank correlation coefficients between hyperglycemic hormonal responses and metabolic parameters for all participants (n = 53) stratified by glycemic status

Hyperglycemic clamp												
AUC	Glucagon			Cortisol			ACTH			GH		
Glycemic status	T2D	PD	NG	T2D	PD	NG	T2D	PD	NG	T2D	PD	NG
n	19	18	16	19	18	16	19	18	16	19	18	16
BMI	0.11	0.36	−0.17	−0.26	−0.19	0.06	0.30	0.29	0.42	−0.05	0.08	**−0**.57*
WHR	**0**.58*	0.09	0.15	0.03	−0.11	−0.06	0.30	0.15	**0**.52*	−0.29	**−0**.49*	**−0**.60*
Body fat	−0.28	0.17	−0.21	−0.06	−0.06	0.06	−0.03	−0.08	0.15	0.12	0.26	−0.28
Fasting glucose	**0**.46*	−0.24	0.06	0.13	**−0**.54*	−0.28	0.40^†^	−0.11	**0**.53*	**−0**.56*	0.13	−0.34
HbA1c	**0**.75*	−0.33	**0**.73*	0.22	−0.16	0.04	**0**.47*	−0.21	0.15	**−0**.49*	0.38	−0.19
M-value*^a^*	−0.34	−0.43^†^	−0.16	0.04	0.23	−0.28	−0.38	−0.15	**−0**.66*	0.17	0.13	**0**.54*
HOMA-IR	**0**.65*	0.39	0.01	0.06	−0.23	−0.19	**0**.72*	0.04	**0**.59*	**−0**.72*	−0.13	**−0**.61*
**ΔAUC**	Glucagon			Cortisol			ACTH			GH		
BMI	0.18	0.47^†^	0.00	0.08	0.34	0.02	−0.27	**0**.66*	0.22	−0.21	0.29	0.36
WHR	0.31	−0.45^†^	0.07	−0.21	0.05	−0.32	−0.01	−0.04	−0.14	0.18	−0.30	0.12
Body fat	−0.07	**0**.56*	−0.11	0.08	0.33	0.05	−0.29	0.36	0.31	−0.24	0.34	0.12
Fasting glucose	0.34	−0.04	**0**.58*	−0.17	−0.43^†^	**−0**.51*	−0.34	−0.09	−0.23	0.13	0.10	−0.01
HbA1c	0.31	0.28	0.27	0.06	−0.29	−0.26	0.36	0.18	−0.49^†^	0.18	−0.02	−0.49^†^
M-value*^a^*	−0.36	−0.09	−0.43^†^	−0.36	−0.30	−0.15	0.15	0.13	0.04	−0.10	0.13	−0.34
HOMA-IR	0.27	−0.08	0.14	0.03	−0.07	−0.12	−0.17	−0.12	−0.10	−0.02	0.21	0.20

ΔAUC in %. Significant coefficients are **bold**.

Abbreviations: ACTH, adrenocorticotropic hormone; AUC, area under the curve; BMI, body mass index; GH, growth hormone; HbA1c, glycated hemoglobin; HOMA-IR, homeostatic model assessment for insulin resistance; NG, normoglycemia; PD, prediabetes; T2D, type 2 diabetes; WHR, waist-hip ratio.

^*a*^Data missing for 1 participant in T2D.

^*^ *P <* .05.

^†^ *P <* .10.

**Table 5.** Multilinear regressions between hormonal responses and metabolic parameters for all participants

Hypoglycemic clamp							
		Glucagon	Cortisol		ACTH		GH
Measure		AUC	AUC	ΔAUC	AUC	ΔAUC	AUC
Model *R^2^*		0.283	0.368	0.270	0.409	0.290	0.280
Model *P*		.003	<.001	.004	<.001	.005	.003
n		53	53	53	53	53	53
WHR	*β*	.041	−.209	.300	.204	.216	NA
Body fat	*β*	NA	NA	NA	NA	NA	−.231
Fasting Glucose	*β*	NA	−.085	NA	−.075	−.030	**−.362** *
M-value	*β*	−.292^†^	**−.544** **	−.115	**−.393** *	**−**.340*	NA
**Hyperglycemic clamp**							
		**Glucagon**		**ACTH**		**GH**	
Measure		AUC	ΔAUC	AUC	ΔAUC	AUC	ΔAUC
Model *R^2^*		0.538	0.680	0.134	0.222	0.378	0.059
Model *P*		<.001	<.001	.140	.015	<.001	.558
n		52	52	52	53	52	53
BMI	*β*	NA	.205^†^	NA	.262^†^	NA	.212
WHR	*β*	.248	NA	−.008	NA	−.317	NA
Fasting glucose	*β*	NA	NA	NA	NA	−.143	NA
HbA1c	*β*	**.660** ***	**.587** ***	NA	.047	NA	NA
M-value	*β*	.038	−.172	−.248	NA	.161	NA
HOMA-IR	*β*	NA	NA	NA	NA	NA	.009

ΔAUC in % in hyperglycemic clamp. Model with best fit, adjusted for age and sex, presented. No model generated for ΔAUC of glucagon and GH in hypoglycemic clamp or Cortisol AUC or ΔAUC in hyperglycemic clamp due to not fulfilling criteria as described in the methods section. Significant coefficients are **bold**.

Abbreviations: ACTH, adrenocorticotropic hormone; AUC, area under the curve; *β*, standardized beta coefficient; BMI, body mass index; GH, growth hormone; HbA1c, glycated hemoglobin; HOMA-IR, homeostatic model assessment for insulin resistance; NA, not applicable, ie, not included in models; WHR, waist-hip ratio.

^†^ *P* < .10.

^*^ *P* < .05.

^**^ *P* < .01.

^***^ *P* < .001.

In brief summary and with minor variations, augmented responses of glucagon (AUC only in hypoglycemic clamp), cortisol, and ACTH and attenuated responses of GH in both clamps correlated with unfavorable metabolic phenotypes, such as adiposity, chronic hyperglycemia, and insulin resistance. Correlations were somewhat differential depending on glycemic status. In particular, glycemic control parameters displayed opposed negative correlations with hypoglycemic responses of glucagon and cortisol in PD, compared to T2D and NG. Moreover, correlations between hypoglycemic cortisol and ACTH responses and measures of adiposity and insulin resistance were not as strong in T2D and PD, compared to NG.

In the final regression models, M-value was overall more strongly associated (negative coefficients) with glucagon (AUC), cortisol, and ACTH responses to hypoglycemia than other metabolic parameters, although the coefficient was not significant with regard to glucagon AUC. Fasting glucose was most strongly, and inversely, associated with GH AUC during hypoglycemia. HbA1c was most strongly, and inversely, associated with glucagon lowering during hyperglycemia. Of note, adiposity measures were also strongly associated with the hormonal responses above. For example, BMI was independently associated with GH AUC during hypoglycemia (*R^2^* = 0.167, model *P* = .010, std *β* = −.308, *P* = .028, fasting glucose also included in model) and glucagon ΔAUC (*R^2^* = 0.652, model *P* < .001, std *β* = .255, *P* = .022, HbA1c and M-value also included in model) during hyperglycemia. When adjusting for sex (and age), these association were generally attenuated below the significance levels, however. None of the metabolic parameters were independently associated with responses of cortisol, ACTH, and GH to hyperglycemia in the final models.

## Discussion

**Figure 5.**
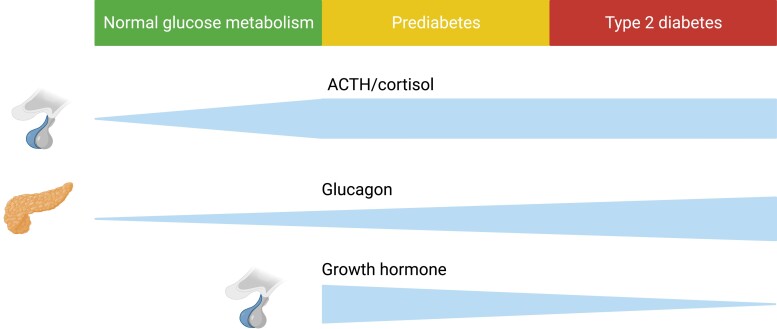
Postulated trajectory of altered insulin-antagonistic hormones in the development of type 2 diabetes. Created with BioRender.com.

In this study, we investigated counter-regulatory hormonal responses to glycemic variations across a large range in individuals with and without T2D. For the first time, comprehensive and detailed investigations using normo-, hypo-, and hyperglycemic clamps were performed in groups representing different stages of T2D development. We demonstrated that different stages of T2D development are characterized by generally upregulated glucagon levels and downregulated levels of GH as well as by increased glucose-dependent responses of the HPA axis. Elevated HPA axis reactivity to glucose lowering, even without overt hypoglycemia, seems to be driven by ACTH, and it was tightly linked to insulin resistance and present already in individuals with prediabetes, thus potentially contributing to T2D development early on. By contrast, chronic hyperglucagonemia, impaired hyperglycemia-induced suppression of glucagon and generally suppressed GH levels seem to be hallmarks of manifest T2D, thus culminating later in its development. These findings position altered secretion of counter-regulatory hormones at different stages of T2D development (as illustrated in Fig. 5), suggesting a mechanistic role which may promote insulin resistance and chronic hyperglycemia and, to some extent, also adiposity.

### Glucagon

Glucagon levels were clearly elevated with an attenuated relative suppression in the normo- and hyperglycemic range for participants with vs without T2D, which seemed to be best explained by compromised glycemic control. In the hypoglycemic range, this group difference gradually diminished. In the summary assessment of the hypoglycemic clamp, there was no group difference between participants with and without T2D, but insulin resistance and central adiposity correlated with elevated glucagon levels. As visualized in Fig. 4B, insulin resistant participants not only had higher glucagon levels throughout most of the hypoglycemic clamp, but also seemed to have an earlier peak of glucagon, resulting in a numerically, albeit nonsignificantly higher glucagon ΔAUC in this clamp. Cellular mechanisms underlying these findings have not been investigated presently, but they may include intra-islet insulin resistance (16), altered glucose sensitivity of alpha cells (17), and hepatic glucagon resistance (18). The hyperglucagonemia currently observed in individuals with T2D and prediabetes agrees well with previous findings by other researchers (19-21). Hypoglycemia-induced responses in relation to previous studies will be discussed further below.

### HPA Axis

HPA axis dynamic responses and hormonal levels did not differ between subjects with T2D and sex-, age-, and BMI-matched subjects without T2D. However, in the full cohort, participants with prediabetes and T2D had augmented hypoglycemic HPA axis responses in comparison to nonmatched participants with normoglycemia, which was independently associated with insulin resistance rather than dysglycemia in multilinear regressions. Notably, correlations between insulin resistance and HPA axis responses were particularly strong among participants with normoglycemia. In general, HPA axis activity displayed less variation due to clinical characteristics in the normo- and hyperglycemic range. These findings support a contribution of intermittent HPA axis overactivity to insulin resistance, even before manifest dysglycemia, that can promote T2D development. However, it is not further aggravated when T2D becomes established, suggesting that the HPA axis may be a pharmaceutical target for prevention rather than treatment of T2D. These data are compatible with previous findings by us and others in individuals without T2D (22-24) and suggest a central HPA axis upregulation mediated via ACTH hypersecretion. Obesity-induced hypothalamic resistance to glucocorticoid inhibition (25) or higher CRH-responsivity to stress (26, 27) may explain these observations. In a recent study, longitudinal increments in other features of HPA axis regulation, such as wake-up cortisol and overall diurnal levels, were linked to increases in fasting glucose in individuals with but not without diabetes (28). Thus, it is possible that disrupted diurnal HPA axis dynamics drive dysglycemia at later stages of T2D development, while elevated HPA reactivity to glucose lowering, and potentially other stress stimuli, is present at earlier stages. Conversely, the reversal of obesity and insulin resistance by gastric bypass surgery is preceded by attenuated HPA as well as glucagon responses to glucose lowering (29), which could contribute to the antidiabetic effects of this intervention.

### Growth Hormone

In sharp contrast to other counter-regulatory hormones, GH levels were generally attenuated in participants with vs without T2D and lower levels of GH correlated with unfavorable metabolic phenotypes. This was observed throughout the investigated glycemic range and is in keeping with previous findings by us and others in individuals without T2D, in which GH levels and dynamics are attenuated in overweight individuals (12, 30). Inhibitory effects of free fatty acids, insulin, and insulin-like growth factor 1 (IGF-1), occurring at the hypothalamic or pituitary level, have been proposed to explain these observations (31). In individuals with T2D, previous results are conflicting, and variations have been attributed to obesity markers (32), which is not quite supported by the present work, where multilinear regressions indicated a greater importance of dysglycemia *per se*. Notably, these models were adjusted for sex, and adiposity markers were stronger predictors in unadjusted models. Hence, sex differences in hypoglycemic counter-regulation and body composition—men have augmented GH responses (33), higher waist-hip ratio, and lower body fat % (34)—could explain the link between adiposity measures and GH levels during hypoglycemia, in particular the paradoxically opposed impact of waist-hip ratio and body fat % observed presently.

In contrast to the detrimental effects of glucagon and cortisol on glucose metabolism, the role of GH is more ambiguous, and it has both insulin sensitizing and desensitizing effects. Further, the former may also be promoted by the downstream release of IGF-1 and the net effects are therefore unclear. Interestingly, correcting the relative GH deficiency in obese individuals with T2D has been reported to improve insulin sensitivity (35, 36). Thus, GH deficiency may also be a cause rather than a consequence of metabolic disturbances.

It should be pointed out that possible interactions between the investigated hormonal systems, such as a stimulating effect of glucagon on ACTH and GH secretion (37), have not been addressed in this study. Therefore, we cannot exclude the possibility that such interactions may be involved in the reported alterations.

### Comparison With Other Studies of Hypoglycemic Counter-Regulatory Responses in T2D

A few previous studies have, although not consistently, demonstrated higher setpoints for, and in some studies higher magnitude of, counter-regulatory hormonal responses to hypoglycemia in patients with T2D vs healthy controls (38-42) that have normalized following metabolic improvement (38). In a cohort resembling the one in the current study, Spyer et al demonstrated a general upward shift in the glycemic setpoint for the response of hormones, including glucagon, cortisol, and GH, in participants with well-controlled T2D (41). In the current study, we found altered magnitudes of these hormone responses, but no clear evidence of altered glycemic setpoints. Most importantly, participants with T2D in our study had better glycemic control and the participants without T2D were less healthy from a metabolic perspective, being predominantly obese, insulin resistant, and having fasting glucose in the range of prediabetes.

The novelty of the present study is the identification of altered regulation in specific counter-regulatory hormonal systems at different stages in the development of T2D, both early and late. Further, we suggest that these alterations contribute to the development of T2D by driving insulin resistance and thereby promoting dysglycemia.

### Strengths and Limitations

In comparison with previous studies, this sample size was large, consisting of participants with a continuum of glycemic dysregulation. Thus, detailed dissection of potential determinants of hormonal responses within and across different subgroups has been possible. Furthermore, the combination of normo-, hypo-, and hyperglycemic clamps has permitted investigations of hormonal responses through a wide glycemic range, about 2.5 to 19 mmol/L in these individuals. There are, however, also limitations that need to be addressed. First, the study procedures did not include an oral glucose tolerance test (OGTT) for classification of T2D and prediabetes, but fasting glucose in combination with HbA1c are considered acceptable measures (43). Second, the participants with T2D generally had good control of glycemia and other vascular risk factors, and they took more chronic medications compared to participants without T2D. Although drugs with known neuroendocrine effects were not allowed in this study, we cannot exclude lingering effects from other agents, such as metformin, antihypertensives, and statins. Third, glucose levels during clamps varied slightly between subject groups, and insulin levels also displayed differences that were, not unexpectedly, most evident during hyperglycemic clamps. This was most likely due to metabolic factors, such as insulin resistance and clearance, as well as beta-cell function. Notwithstanding the performed adjustments for ambient glucose levels and other factors, they may have affected some results. Fourth, the study population exhibited considerable dispersion with regard to age and BMI, but it was predominantly female, relatively young (in particular in the NG group), and overweight/obese. As a result, the interpretability and generalizability of the results may be somewhat limited. Fifth, activity in the HPA and GH axes vary considerably during the day and we cannot exclude the possibility that the experiments would have yielded different results, had they been scheduled at a different time of the day. Sixth, hypo- and hyperglycemic clamps are not physiological, but nonetheless, the current findings were observed at glycemic levels that were largely within a reasonable physiologic range, as judged by continuous glucose monitoring (CGM) studies indicating a nonnegligible prevalence of biochemical hypoglycemia and hyperglycemia in individuals with normal glucose tolerance as well as prediabetes (44, 45). Finally, we acknowledge that the current cross-sectional study design does not permit firm conclusions regarding causality between altered neuroendocrine regulation and the development of T2D. Additional analyses of autonomic nervous system, catecholamine, and lipolysis responses are planned in the current cohort. Future research should aim to further elucidate the causal role of neuroendocrine regulation, for example, by targeted clinical intervention studies or by longitudinal follow-up of individual trajectories along the gradual progression to prediabetes and T2D, including repeated assessments of counter-regulatory biomarkers.

## Conclusions

Type 2 diabetes development is associated with elevated glucagon levels—both due to chronic hypersecretion but also to impaired suppression by hyperglycemia—and downregulated GH levels. The alterations in these two hormonal systems culminate late, once type 2 diabetes is established. By contrast, elevated HPA axis reactivity to glucose lowering appears to be an early feature that is linked more strongly to insulin resistance and central adiposity than to hyperglycemia and type 2 diabetes. We propose that altered neuroendocrine regulation, largely brain-mediated, promotes insulin resistance and contributes to the development of type 2 diabetes at different stages.

## Data Availability

Some or all datasets generated during and/or analyzed during the current study are not publicly available but can be provided from the corresponding author on reasonable request.

## Abbreviations

ACTH: adrenocorticotropic hormone
AUC: area under the curve
BMI: body mass index
CV: coefficient of variation
FFM: fat-free mass
GH: growth hormone
HbA1c: glycated hemoglobin
HOMA-IR: homeostatic model assessment for insulin resistance
HPA: hypothalamic-pituitary-adrenal
IQR: interquartile range
ND: nondiabetic
NG: normoglycemia
PD: prediabetes
T2D: type 2 diabetes
